# Effects of regenerative peripheral nerve interface on dorsal root ganglia neurons following peripheral axotomy

**DOI:** 10.3389/fnins.2022.914344

**Published:** 2022-09-07

**Authors:** Zheng Wang, Dong Zhang, Xin Zeyu Yi, Yong Zhao, Aixi Yu

**Affiliations:** Department of Orthopedics Trauma and Microsurgery, Zhongnan Hospital of Wuhan University, Wuhan, China

**Keywords:** RPNI, dorsal root ganglion, nerve injury, neurotrophins, neuroprotection, neuronal loss

## Abstract

**Background:**

Long-term delayed reconstruction of injured peripheral nerves always results in poor recovery. One important reason is retrograde cell death among injured sensory neurons of dorsal root ganglia (DRG). A regenerative peripheral nerve interface (RPNI) was capable of generating new synaptogenesis between the proximal nerve stump and free muscle graft. Meanwhile, sensory receptors within the skeletal muscle can also be readily reinnervated by donor sensory axons, which allows the target muscles to become sources of sensory information for function reconstruction. To date, the effect of RPNI on injured sensory neurons is still unclear. Here, we aim to investigate the potential neuroprotective role of RPNI on sensory DRG neurons after sciatic axotomy in adult rats.

**Materials and methods:**

The sciatic nerves of sixty rats were transected. The rats were randomly divided into three groups following this nerve injury: no treatment (control group, *n* = 20), nerve stump implantation inside a fully innervated muscle (NSM group, *n* = 20), or nerve stump implantation inside a free muscle graft (RPNI group, *n* = 20). At 8 weeks post-axotomy, ipsilateral L4 and L5 DRGs were harvested in each group. Toluidine blue staining was employed to quantify the neuronal densities in DRGs. The neuronal apoptosis index was quantified with TUNEL assay. Western blotting was applied to measure the expressions of Bax, Bcl-2, and neurotrophins (NTs) in ipsilateral DRGs.

**Results:**

There were significantly higher densities of neurons in ipsilateral DRGs of RPNI group than NSM and control groups at 8 weeks post-axotomy (*p* < 0.01). Meanwhile, neuronal apoptosis index and the expressions of pro-apoptotic Bax within the ipsilateral DRGs were significantly lower in the RPNI group than those in the control and NSM groups (*p* < 0.05), while the opposite result was observed in the expression of pro-survival Bcl-2. Furthermore, the expressions of NGF, NT-3, BDNF, and GDNF were also upregulated in the ipsilateral DRGs in the RPNI group (*p* < 0.01).

**Conclusion:**

The present results demonstrate that RPNI could prevent neuronal loss after peripheral axotomy. And the neuroprotection effect has a relationship with the upregulation of NTs in DRGs, such as NGF, NT-3, BDNF, and GDNF. These findings provide an effective therapy for neuroprotection in the delayed repair of the peripheral nerve injury.

## Introduction

Immediate repair is the ideal target for nerve reconstruction after peripheral nerve injury, but this is not possible and delayed repair is required on many occasions, such as long nerve defect, severe local infection, or poor tissue conditions (Wu et al., 2013). However, no matter how well the nerve gap is surgically repaired, long-term delayed nerve repair often results in poor recovery (Desouches et al., 2005; George and Boyce, 2014). Apart from prolonged denervation of distal nerve stumps and target muscles (Gordon et al., 2011), one important reason is retrograde cell death among injured sensory neurons of the dorsal root ganglia (DRG), which will restrict the capacity for peripheral nerve regeneration and usually results in severely disabling sensory deficits (Terenghi et al., 2011). Poor sensory outcomes will also have detrimental impacts on motor function, especially in fine manipulative work (Schenker et al., 2006), since adequate sensory feedback for normal motor control is warranted (Westling and Johansson, 1984).

Retrograde cell death can occur in both DRG and spinal cord neurons after peripheral nerve transection or limb amputation. But it is generally agreed that spinal motor neurons are more resistant than primary sensory neurons. The death of spinal motor neurons occurs mainly when the nerve is severed near the spinal cord, such as ventral root avulsion (Hoffmann et al., 1993; Gu et al., 1997; Ma et al., 2001). By contrast, sensory neurons in DRG are more susceptible to die, even if the transection occurs at the distal peripheral nerve (Wilson et al., 2003; Hart et al., 2008; Terenghi et al., 2011). In the adult animal model of sciatic nerve division at the upper border of quadratus femoris, neuronal death begins within 24 h of peripheral axotomy and reached a peaked value of 21% at 2 weeks after injury. The majority of neuronal loss, about 35%, occurs within the first 2 months and limited death is still occurring at 6 months (Wilson et al., 2003).

Although the injury-signaling pathways involved in triggering the intrinsic regeneration capacity of injured sensory neurons are not fully understood, it is generally accepted that impaired retrograde transport of target-derived neurotrophins (NTs) plays a determinant role in this process (Ambron and Walters, 1996; Fu and Gordon, 1997; Cui, 2006; Li et al., 2020). It has been shown that neuronal loss could be rescued by the local administration of exogenous NTs, such as the nerve growth factor (NGF), neurotrophin-3 (NT-3), neurotrophin-4 (NT-4), brain-derived neurotrophic factor (BDNF), and glial cell line-derived neurotrophic factor (GDNF) (Hart et al., 2008). However, the clinical use of these exogenous NTs is hampered by the continuous intrathecal administration, potential carcinogenic actions or toxicity (Hart et al., 2002). Apart from NTs, Acetyl-L-carnitine (ALCAR) and N-acetyl-cysteine (NAC), two clinically safe pharmaceutical agents with antioxidant effects (Ezeriņa et al., 2018; Di Emidio et al., 2020), also exhibit potential neuroprotective properties (Ferreira and McKenna, 2017; Tardiolo et al., 2018; Chakraborty et al., 2020; Pennisi et al., 2020), and probably can rescue neurons from retrograde degeneration (Hart et al., 2002; Wilson et al., 2003; Welin et al., 2009). But further preclinical studies are still required before a systematic clinical application. Therefore, it is urgent to develop a clinically feasible and safe therapy that will prevent sensory neuron death after peripheral nerve transection.

Recently, the regenerative peripheral nerve interface (RPNI) was reported as a practical and reproducible surgical procedure to transduce and amplify neural signals for the purpose of controlling a neuroprosthetic limb (Kung et al., 2014; Frost et al., 2018; Vu et al., 2020). An RPNI is constructed by implanting the proximal nerve stump (PNS) into a free skeletal muscle graft, and these muscle fibers subsequently serve as denervated targets for regenerating axons sprouting from the PNS (Ursu et al., 2016; Kubiak et al., 2018; Vu et al., 2020). The RPNI is able to generate compound muscle action potentials of substantive amplitude that can be recorded from a transcutaneous electrode as early as 1 month after implantation, due to the formation of new neuromuscular junctions (synaptogenesis) within the muscle graft (Kung et al., 2014; Ursu et al., 2016; Vu et al., 2020). Apart from the formation of functional neuromuscular junctions, sensory receptors (Golgi tendon organs and spindle cells) within the skeletal muscle can also be readily reinnervated by donor sensory axons, which allows the target muscles to become sources of sensory information for function reconstruction (Vu et al., 2020).

A recent study has demonstrated that targeted muscle reinnervation (TMR), another neural-machine interface technology that involves transferring the PNS into specific target muscles, could improve the spinal cord microenvironment for the survival of motor neurons (Lu et al., 2022). However, the effect of RPNI on injured neurons is still unclear. Considering that the sensory DRG neurons are more susceptible after distal peripheral nerve transection, we aimed to investigate the potential neuroprotective role of RPNI on sensory DRG neurons after sciatic axotomy in an adult rat model.

## Materials and methods

### Animal models and grouping

All experiments were carried out in strict accordance with the Guide for the Care and Use of Laboratory Animals prepared by the National Institutes of Health and approved by the Experimental Animal Welfare Ethics Committee of Zhongnan Hospital of Wuhan University under animal protocol number ZN2021094. A total of sixty male SD rats, weighing 200–250 g, were purchased from the Experimental Animal Center of Wuhan University (Wuhan, China). Animals were kept in groups of three in plastic cages with floor covered with soft bedding at 25°C and maintained on a light/dark cycle of 12 h day/night. Food and water were made available *ad libitum*. The rats were randomly divided into three groups: no treatment (control group, *n* = 20), nerve stump implantation inside a fully innervated muscle (NSM group, *n* = 20), or nerve stump implantation inside a free muscle graft (RPNI group, *n* = 20).

### Surgical procedures

**FIGURE 1:**
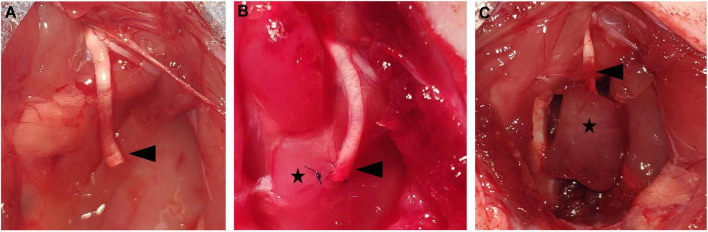
The presentation of the operations in each group. **(A)** The control group. The sciatic PNS (black arrowhead) was left *in situ* without treatment. **(B)** The NSM group. The sciatic PNS (black arrowhead) was embedded in the nearby adductor magnus muscle (black star). **(C)** The RPNI group. The sciatic PNS (black arrowhead) was implanted inside the free muscle graft (black star). DRG, dorsal root ganglia; NSM, nerve stump implantation inside the muscle; PNS, proximal nerve stump; RPNI, regenerative peripheral nerve interface.

The rats were deeply anesthetized using intraperitoneal pentobarbital sodium (1%, 5 mL/kg; Sigma-Aldrich, St. Louis, MO, United States). The left sciatic nerve was exposed *via* posterior thigh approach and sharply transected 1 cm from the original branch of the posterior gluteal nerve. Then, an 1–1.5 cm nerve defect was made to prevent spontaneous reinnervation. In the control group, the PNS was left *in situ* with no treatment (Figure 1A). In the NSM group, the PNS was implanted into a muscle sac that was made through a small incision along the direction of the muscle fibers on the nearby adductor Magnus muscle. The epineurium of the sciatic nerve was fixed with the epimysium by two 10-0 nylon sutures (Figure 1B). In the RPNI group, a free muscle graft measuring approximately 1.0 cm × 0.5 cm × 0.3 cm was harvested from the nearby Adductor Magnus muscle along the direction of the muscle fibers. The PNS was placed at the middle of the muscle graft, paralleled to the muscle fibers, and secured distally with 10-0 nylon sutures in an epimysium-to-epineurium fashion. Then, wrapping the muscle graft edges around the PNS and securing with 7-0 nylon sutures completes the construction of the RPNI (Figure 1C). Finally, the left leg incisions were closed in layers with 4-0 sutures in all groups. Once recovered from anesthesia, animals were injected subcutaneously with a meloxicam analgesic (0.2 mg/mL/kg; Boehringer-Ingelheim, Mainz-Bingen, Germany).

### Neuron counting

The ipsilateral L4 DRGs were harvested and processed for neuron counting with toluidine blue staining at 8 weeks after surgery (*n* = 5). The specimens were fixed in 4% paraformaldehyde at 4°C and then cut into 5 μm sections transversely. To avoid double counting of the number of neurons, each section was cut at a 50 μm interval. Then, 20 sections were randomly selected from each group for subsequent toluidine blue staining. Briefly, the sections were stained with 1% toluidine blue (Baiqiandu, Wuhan, China) for 20 min. Then, they were treated with alcohol for rapid color separation and permeabilized with xylene. As described previously (Kryger et al., 2001), the DRGs were evaluated quantitatively by comparing neuron density. Five sample sites per sections were randomly selected at 2 mm intervals in the microscope stage. The total number of DRG neurons was counted blindly in fields using a square grid in the eyepiece. The local densities were converted into neurons/mm^2^.

### TUNEL staining

To demonstrate the anti-apoptotic effects of RPNI, TUNEL assay for ipsilateral L4 DRGs was performed at 8 weeks post-axotomy (*n* = 5), according to the manual of the Roche TUNEL kit. Briefly, sections (5 μm) were incubated for 30 min at 37°C with proteinase K (Baiqiandu) working solution. After being washed with PBS for three times, the sections were stained with the antibodies and then subjected to TUNEL staining using Cell Death Detection kit (Roche, Basel, Switzerland). In the end, the sections were covered with a mounting medium containing DAPI and visualized using a fluorescent microscope (Olympus, Tokyo, Japan). And five visual fields were counted to calculate TUNEL positive cells. The apoptosis index (AI) was calculated using the following equation: AI = (number of TUNEL-positive neurons/total neuron number [DAPI]) × 100%.

### Western blot analysis

Western blot analysis was employed to evaluate the expression of Bcl-2, Bax, NGF, NT-3, BDNF, and GDNF from pooled ipsilateral L4-L5 DRGs at 8 weeks post-surgery (*n* = 5). Equal amounts of proteins from specimens were loaded onto a 5% sodium dodecyl sulfate-polyacrylamide gel electrophoresis and transferred to polyvinylidene fluoride membranes (Millipore, Billerica, MA, United States). The membranes were blocked in 5% bovine serum albumin (Solarbio, Beijing, China) for 2 h at room temperature and then incubated with primary antibodies against the target proteins over night at 4°C. The relevant information about all primary antibodies was listed in Table 1. The membranes were washed in tris buffered saline tween (TBST; Baiqiandu) for three times and then incubated with the secondary antibody, horseradish peroxidase-conjugated goat anti-rabbit IgG (dilution 1:50000, 074-1506, KPL, Gaithersburg, MD, United States) or horseradish peroxidase-conjugated goat anti-mouse IgG (dilution 1:50000, 074-1806, KPL) at room temperature for 30 min. Membranes were rewashed in TBST for three times, and labeled protein bands were visualized using an Enhanced Chemiluminescence Detection Kit (Baiqiandu) and analyzed with ImageJ software (National Institutes of Health, Bethesda, MD, United States). The relative levels of the target proteins were normalized to β-actin.

**TABLE 1:** The relevant information of the primary antibodies.

Name	Species	Manufacturer	Article number	Dilution
Bax	Rabbit	Proteintech, United States	50599-2-Ig	1:5000
Bcl-2	Mouse	Proteintech, United States	60178-1-Ig	1:5000
NGF	Rabbit	ABclonal, United States	A14216	1:1000
NT-3	Rabbit	Proteintech, United States	18084-1-AP	1:2000
BDNF	Rabbit	HuaBio, China	ET1606-42	1:2000
GDNF	Rabbit	ABclonal, United States	A14639	1:1000
β-actin	Rabbit	Abcam, United States	ab227387	1:6000

### Statistical analysis

All relevant data were presented as mean ± SD and analyzed by GraphPad Prism7 (GraphPad Software, San Diego, CA, United States). Differences between the three groups were evaluated using one-way analysis of variance followed by Tukey’s *post-hoc* test. Statistical significance was assigned for *p*-value < 0.05.

## Results

### Neuron counting

**FIGURE 2:**
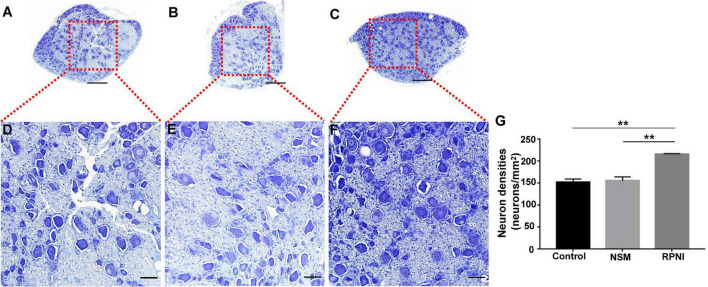
The results of neuron counting in the ipsilateral L4 DRG among the three groups at 8 weeks post-axotomy. **(A–F)** Toluidine blue-stained neuronal cell bodies within the ipsilateral L4 DRG in the control **(A,D)**, NSM **(B,E)** and RPNI **(C,F)** groups. Regions marked with red boxes in panels **(A–C)** are depicted at a higher magnification in panels **(D–F)**. **(G)** Neuron densities of the ipsilateral DRGs in each group. [**p < 0.01. Scale bar = 100 μm **(A–C)**, Scale bar = 25 μm **(D–F)**]. DRG, dorsal root ganglia; NSM, nerve stump implantation inside the muscle; RPNI, regenerative peripheral nerve interface.

Morphologically, the appearance of the neuronal cell bodies within the ipsilateral DRGs taken from the three groups was indistinguishable (Figures 2A–F). At 8 weeks after axotomy, the neuron densities in axotomized ipsilateral L4 DRGs from the RPNI group was 215.7 ± 1.122 neurons/mm^2^, much higher than 155.8 ± 4.952 neurons/mm^2^ in the NSM group and 152.9 ± 4.057 neurons/mm^2^ in the control group (both *p* < 0.01) (Figure 2G). In contrast, there was no statistically significant difference between the control and NSM groups (*p* > 0.05).

### TUNEL assay

**FIGURE 3:**
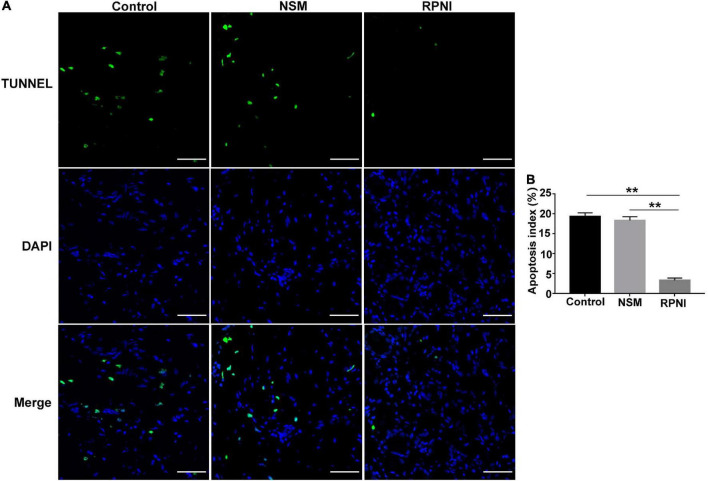
The results of TUNEL assay in the ipsilateral L4 DRGs among the three groups at 8 weeks post-axotomy. **(A)** Fluorescent images of neurons in the ipsilateral L4 DRG taken from each group. **(B)** Apoptosis index (TUNEL-positive neurons/total neuron number [DAPI]) in each group. (**p < 0.01. Scale bar = 50 μm). DAPI, 4′,6-Diamidine-2′-phenylindole dihydrochloride; DRG, dorsal root ganglia; NSM, nerve stump implantation inside the muscle; RPNI, regenerative peripheral nerve interface.

Apoptotic neurons in the DRGs were detected as TUNEL-positive cells (Figure 3). RPNI could decreased neuronal apoptosis, resulting that the apoptosis index in the DRGs of RPNI group was lower than the control and NSM groups (both *p* < 0.01). However, there was no significant difference in the apoptosis index between the control and NSM groups (*p* > 0.05).

### Expressions of apoptotic-related markers in ipsilateral dorsal root ganglias

**FIGURE 4:**
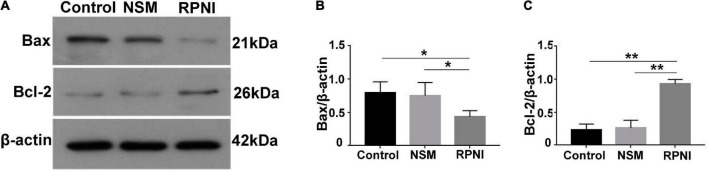
Western blot results of apoptosis-related proteins in ipsilateral DRG. **(A)** Western blotting bands of Bax and Bcl-2 expressions from pooled ipsilateral L4 and L5 DRGs harvested at 8 weeks after surgery. **(B,C)** The quantifications of corresponding western blot signals in panel **(A)**. (*p < 0.05, **p < 0.01). DRG, dorsal root ganglia; NSM, nerve stump implantation inside the muscle; RPNI, regenerative peripheral nerve interface.

The expression levels of apoptotic markers, including Bax and Bcl-2 were tested by western blot analysis. As shown in Figures 4A–C, an increased Bax and decreased Bcl-2 expressions were observed in RPNI group at 8 weeks post-axotomy compared to the control group (both *p* < 0.05). These results demonstrated that RPNI could significantly attenuate the neuronal apoptosis induced by axotomy. However, there was no significant difference between the NSM and control groups (*p* > 0.05).

### Expressions of neurotrophins in ipsilateral dorsal root ganglias

**FIGURE 5:**
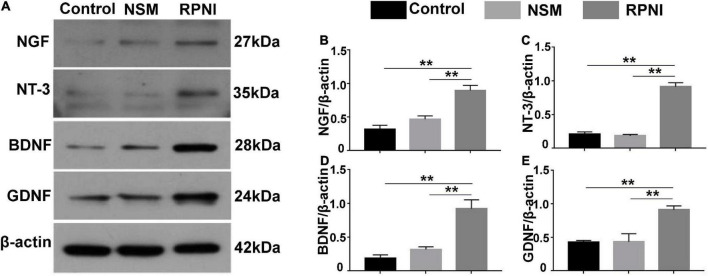
Western blot results of NTs in ipsilateral DRG. **(A)** Western blotting bands of NGF, NT-3, BDNF, and GDNF expressions from pooled ipsilateral L4 and L5 DRGs harvested at 8 weeks after surgery. **(B–E)** The quantifications of corresponding western blot signals in panel **(A)**. (**p < 0.01). BDNF, brain-derived neurotrophic factor; DRG, dorsal root ganglia; GDNF, glial cell line-derived neurotrophic factor; NSM, nerve stump implantation inside the muscle; NGF, nerve growth factor; NT-3, neurotrophin-3; NTs, neurotrophins; RPNI, regenerative peripheral nerve interface.

As shown in Figures 5A–E, the expression levels of NTs from ipsilateral DRGs, including NGF, GDNF, BDNF, and NT-3 in the RPNI group were statistically increased compared to those in the control group (all *p* < 0.01). However, there was no significant difference between the NSM and control groups (*p* > 0.05).

## Discussion

The retrograde cell loss among injured sensory DRG neurons always results in poor recovery after long-term delayed reconstruction of injured peripheral nerves. However, effective methods to prevent neuronal loss are still absent. RPNI, a neuroprosthesis controlling technology, can form a functional connection between the PNS and a free muscle after amputation. Herein, we firstly found that RPNI could rescue the loss and apoptosis of sensory DRG neurons induced by sciatic nerve transection. Moreover, the expressions of multiple NTs in ipsilateral DRG including NGF, BDNF, NT-3, and GDNF were increased after RPNI surgery, which might be involved in the process of RPNI protecting sensory DRG neurons.

The peripheral nerve axons have both anterograde and retrograde axonal transport functions (Sleigh et al., 2020). Target organs of neurons like muscles can synthesize a large number of NTs, such as NGF, BDNF, NT-3, and GDNF, which can be retrogradely transported to neuron cell bodies for supporting neuron survival (DiStefano et al., 1992; Chen et al., 2002; Wehrwein et al., 2002; Delezie et al., 2019). Once the axon breaks, these target-derived NTs cannot reach neurons, which appears to be the most significant determinant of neuron loss in peripheral nerve injury (Ambron and Walters, 1996; Fu and Gordon, 1997; Cui, 2006; Li et al., 2020). Therefore, restoring retrograde axonal transport is essential for the survival of proximal neurons. A recent study has demonstrated that another neural-machine interface technology TMR, which involves transferring the PNS into specific target muscles, could improve the spinal cord microenvironment for the survival of motor neurons (Lu et al., 2022). Actually, RPNI has many similarities with TMR, but is much simpler than TMR (Loewenstein et al., 2021). Once an RPNI is established, synaptogenesis can be formed between the PNS and the free muscle graft as early as 1 month after surgery (Kung et al., 2014; Ursu et al., 2016; Vu et al., 2020). In our study, we found higher densities of ipsilateral DRG neurons in RPNI group than the NSM and control groups at 8 weeks after surgery, which demonstrated that RPNI could prevent axotomy-induced loss of sensory neurons in the DRGs. This effect may attribute to the new connection between the sensory receptors (Golgi tendon organs and spindle cells). In addition, our TUNEL assay found that the neuron apoptosis index in the ipsilateral DRG of the RPNI group was significantly decreased while there was no significant difference between the NSM and control groups. Previous studies showed that neuronal cell apoptosis could be mediated by the pro-survival mediator Bcl-2 and the pro-apoptotic mediator Bax (Zong et al., 2001; Yan et al., 2020). Thus, we chose Bax and Bcl-2 as apoptotic-related markers after neurotomy and found that Bax expression was decreased but Bcl-2 expression was increased in RPNI group. And there was no significant difference in the expressions of Bax and Bcl-2 between the NSM and control groups. These results suggested that RPNI surgery could prevent neuronal loss by inhibiting neuronal apoptosis.

After the sciatic nerve stump is transposed into the target muscle, the neuromuscular junction is established and material transport resumes, which may be crucial for RPNI to improve the microenvironment within the ipsilateral DRG. As mentioned above, upregulation of NTs in the injury site or atomized DRG is expected to reverse the neuron loss, and thus improve the effect of delayed nerve repair since neuronal survival is a prerequisite for axonal regeneration and possible functional recovery after injury [15]. To test whether the inhibition of neuron apoptosis by reinnervated muscle was associated with increased endogenous NTs, we examined the protein expressions of NTs in ipsilateral DRGs among the three groups and found that ipsilateral DRGs expressed higher NGF, NT-3, BDNF, and GDNF in the RPNI group than these in the control group following sciatic axotomy. But there was no significant difference in NTs expression between the NSM and control group. Based on these results, we speculated that the distal reinnervated muscle could probably serve as a physiological “NTs factory” providing endogenous NTs that might be retrogradely transported to neurons in DRG. However, the muscle belly in the NSM group is already fully innervated by its native motor nerve and the regenerating axons of the transected peripheral nerve will not be presented with any targets to reinnervate (Vu et al., 2020), resulting that no NTs could be retrogradely transported and neuronal apoptosis could not be prevented. Although the application of exogenous NT can also benefit neuronal survival, the need of continuous intrathecal administration, potential carcinogenic actions and toxicity in other cell types prevent clinical translation of this method [16]. Moreover, a single kind of NT may sometimes have protective effect on a particular neuronal subpopulation (Hart et al., 2008). Therefore, RPNI would be promising for neuronal protection of multiple subpopulations, because complex cocktail of endogenous NTs could be sustainably released locally and retrogradely transported to neurons without sophisticated methods. Furthermore, the reinnervated muscle might also amplify the production of NTs when it heals with the surrounding muscles.

Several limitations exist in our study. First, these data only suggested a direct link between RPNI and modulating upstream NTs expression in DRG. However, these increased NTs expressions were only suspected to have a relationship with reinnervated muscle by retrograde transport, and whether there were other mechanisms leading to this molecular event was unclear. Another limitation of this study is that the current results only indicated that RPNI could inhibit neuronal loss at 8 weeks after surgery, but the earlier or longer effects of neuron protection by RPNI have not been involved. Therefore, future studies are warrant to reveal the hypothetical link and whether RPNI can prevent neuronal loss in delayed nerve repair at different times.

## Conclusion

In our study, we firstly demonstrated that RPNI, a practical and reproducible surgical solution, could prevent neuronal loss after peripheral axotomy. And the neuroprotection effect may be ascribed to the upregulation of multiple NTs in the ipsilateral DRG, such as NGF, NT-3, BDNF, and GDNF. Although the molecular mechanisms of this approach are not yet fully understood, the neuroprotection profile of RPNI in the animal model evinces its promise for its clinical translation as an effective therapy for neuroprotection in the delayed repair of peripheral nerve transection.

## Data availability statement

The original data presented in this study are included in the article, further inquiries can be directed to the corresponding authors.

## Ethics statement

The animal study was reviewed and approved by the Experimental Animal Welfare Ethics Committee of Zhongnan Hospital of Wuhan University.

## Author contributions

YZ and AY: study design and conception. ZW, DZ, and XY: experiment implementation, data collection and analysis, and manuscript draft. ZW, DZ, YZ, and AY: data interpretation. All authors approved the final version of this manuscript for publication.
